# Distribution of select per- and polyfluoroalkyl substances at a chemical manufacturing plant

**DOI:** 10.1016/j.jhazmat.2023.133025

**Published:** 2023-11-19

**Authors:** Brian A. Schumacher, John H. Zimmerman, Alan C. Williams, Christopher C. Lutes, Chase W. Holton, Elsy Escobar, Heidi Hayes, Rohit Warrier

**Affiliations:** a US EPA Office of Research and Development (ORD), Center for Environmental Measurement & Modeling, 960 College Station Road, Athens, GA 30605, USA; b US EPA ORD, Center for Environmental Measurement & Modeling, 109 T.W. Alexander Drive, Research Triangle Park, NC 27711, USA; c Jacobs, 9304 Coachway, Chapel Hill, NC 27516, USA; d Geosyntec Consultants, 5670 Greenwood Plaza Blvd, Greenwood Village, CO 80111, USA; e Jacobs, 2001 Market Street, Suite 900, Philadelphia, PA 19103, USA; f Eurofins Air Toxics, LLC, 180 Blue Ravine Road, Suite B, Folsom, CA 95630, USA; g Research Triangle Institute, International, 3040 East Cornwallis Road, Research Triangle Park, NC 27709, USA

**Keywords:** PFAS, Perfluorinated carboxylic acids, Fluorotelomer alcohols, Soil, Soil gas, Subslab gas, Groundwater, Vapor intrusion

## Abstract

Per- and polyfluoroalkyl substances (PFAS) are used in various industrial products; however, they pose serious health risks. In this study, soil, soil gas, and groundwater samples were collected at a PFAS manufacturing facility in New Jersey, USA, to determine the presence and distribution of PFASs from the soil surface to groundwater and at various distances from the presumed source. Fluorotelomer alcohols (FTOHs) were detected in soil (< 0.26–36.15 ng/g) and soil gas (160–12,000 E µg/m^3^), while perfluorinated carboxylic acids (PFCAs) were found in soil (4.3–810 ng/g), soil gas (<0.10–180 µg/m^3^), and groundwater (37–49 µg/L). FTOH and PFCA concentrations decreased as the distance from the presumed source increased, suggesting that PFCAs are likely to migrate in groundwater, whereas FTOHs primarily move in the vapor phase. The presence of PFAS in the groundwater, soil, and soil gas samples indicate its potential for vapor intrusion; thus, some PFAS may contribute to indoor air inhalation exposure. To the best of our knowledge, this is the first report on the quantification of volatile PFAS in soil gas at a PFAS manufacturing facility.

## Graphical Abstract

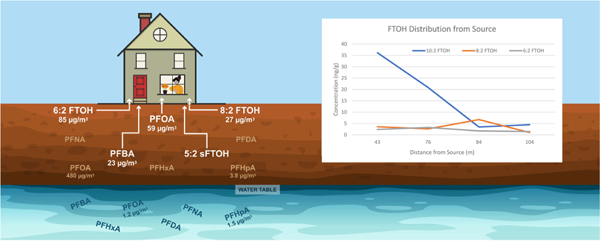

## 1. Introduction

Perfluoroalkyl and polyfluoroalkyl substances (PFAS) are manufactured chemicals that have been used in industrial and consumer products since the 1940s because of their useful properties [50,54]. Various PFAS exist, some of which have been more widely used and studied than others. PFAS have been found in soil and water at or near waste sites, such as landfills [31]; disposal and hazardous waste sites, where aqueous-film-forming foams (AFFFs) are used to extinguish flammable liquid–based fires [36]; manufacturing or chemical production facilities that produce or utilize PFAS; food packaging [11]; household products that are stain and water repellent, cleaning products, non-stick cookware, paints, sealants, personal care products; and biosolids from wastewater treatment plants that are used as fertilizers on agricultural lands (US EPA, 2022a).

Strategies for regulating PFAS concentrations in various environmental matrices, including soil and groundwater, are rapidly being developed in the state and federal jurisdictions [22,51]. The regulatory and guidance values for PFAS vary across federal and state jurisdictions due to different mandates (e.g., regulations and policies), the selection and interpretation of different key toxicity studies, choice of uncertainty factors, approaches used for animal-to-human extrapolation, and exposure assumptions [13,22]. PFAS threshold concentrations that are protective of the groundwater typically range from 0.04 to 6.0 µg/L at the federal level and 0.002–40,000 µg/L at the state level [21]. The PFAS threshold concentrations in the groundwater are lower than those in soil, which typically range, depending on the compound, from 0.000038 to 0.00194 mg/kg and 0.000004–1,000,000 mg/kg at the federal and state levels, respectively [21].

Numerous polyfluorinated organic substances (e.g., fluorotelomer alcohols, FTOHs) have sufficient vapor pressure (>1 mmHg) and Henry’s Law constants (HLCs, >1E-5 atm m^3^/mol) to be designated as vapor-forming chemicals based on the current federal and state vapor intrusion (VI) guidance [1,22]. For reference, 6:2 and 8:2 FTOHs have HLCs of 1.26 and 1.98 atm m^3^/mol, respectively, while perfluorooctanoic acid (PFOA) has an HLC of 3.57 E-6 atm m^3^/mol [22]. From this perspective, vinyl chloride, tetrachloroethylene, and naphthalene have HLCs of 0.0278, 0.0177, and 4.40 E-4 atm m^3^/mol, respectively [34]. According to the technical guide of the U.S. Environmental Protection Agency (USEPA) [48], vapor-forming chemicals should be investigated for vapor intrusion “when they occur as subsurface contaminants at sites subject to a federal statute for land cleanup (e. g., CERCLA, RCRA corrective action)”. The volatility of PFAS is influenced by the non-fluorinated part of its molecules [17]; for example, alcohols are more volatile than carboxylic acids [28]. PFAS with small non-fluorinated structures have been found to be extremely volatile, regardless of its total molecular size [17].

FTOHs have been detected in several consumer and building products, measured in ambient air and surface water [58], indoor air [32, 38], and soil [14,59]. Moreover, FTOH has been modeled to determine its vapor intrusion (VI) potential from the release of AFFFs into the soil [43]. Elevated concentrations of PFAS, including FTOHs, have been quantified in landfill gases and ambient air [16,42]. Early research on FTOH stability in aerobic soils reported that the half-lives of FTOHs only last for a few weeks [55]; however, [35] found that the half-life 8:2 FTOH under sulfate- and iron-reducing conditions lasted for approximately 400 days, indicating the extended FTOH persistence under conditions commonly observed in the soil.

Several PFAS compounds are semi-volatile and partition between atmospheric particulate matter, mists, and the vapor phase [33]. Perfluoroalkyl carboxylic acids (PFCAs) are predominantly partitioned into particulate phases in the atmosphere [22]. Since PFCAs are primarily ionic at environmentally relevant pH, their volatility is expected to be low.

The formation of in situ foams in the soil in the presence of the aqueous solutions of surfactants and gas flows has been reported [10, 30]. [24] reported that PFCAs can be introduced into the atmosphere through bubble bursting. Bastow et al. [9] described how the equilibrium between the ionized and neutral forms of PFAS can be shifted toward neutral PFAS, thus enhancing its volatility when organic phases, such as the diethylene glycol monobutyl ether used in some aqueous fire-fighting foam formulations or the bitumen component of asphalt, are significant components of the matrix.

Numerous researchers have developed sampling and analysis methods for PFAS in ambient air, often involving a filter and PUF/XAD-2 sorbent system [12,2,8]] or PUF disks impregnated with XAD-4 resin [4]. Sorbent collection and thermal desorption methods for PFAS have also been reported [37,40,56].

The authors are not aware of any prior measurements of FTOHs in soil and gas or other studies that directly document the VI potential of these compounds; however, a model suggests that VI could be a concern for certain PFAS compounds [43]. This is the initial research of the USEPA to determine whether the VI exposure pathway is relevant for the selection of PFAS, with a focus on FTOHs, because some PFAS compounds may contribute to inhalation exposure through the VI pathway.

## 2. Materials and methods

### 2.1. Test site

A PFAS manufacturing plant in New Jersey, USA was selected as the test site because it has been producing fluoroelastomers since the late 1950s (Fig. S1). The site is underlain by a sequence of alternating coarse- and fine-grained soil. The primary hydrologic units include a discontinuous A zone (shallowest) and aquifers labeled B–F with intervening aquitards. The groundwater flows across the site in both the horizontal and vertical directions. The saturated groundwater in Zone A is not laterally extensive and discharges to the surface water, where it is not controlled by passive flow barriers or recharges the underlying B aquifer. Groundwater flow in aquifer B is downward to the deeper aquifers C and D or horizontally to the surface water.

Sample locations (A–D) were installed in unvegetated industrial areas at a New Jersey site downgradient from a presumed source building at increasing distances (Fig. S1). Historical reports suggest that previous building foundations may have existed at the planned sampling locations A and D, which could be used to simulate a building slab. Sampling locations A and D were covered with gravel, which was removed before utility clearance and soil gas probe installation. Field observations indicated that the soil beneath the gravel was primarily a mix of coarse- and medium-grained sand with cobbles (Table S1). Location B is a paved parking lot. At location C, a subslab port was installed on the pavement at the edge of the road, whereas the other sampling depths were collected in an adjacent gravelly sand area.

### 2.2. Sample collection

Subslab soil gas was collected from a presumed source building. Soil, soil gas, subslab soil gas, and groundwater (when obtainable) were collected from locations A–D. Soil samples were composite grab samples collected over the full length of the core (i.e., from the surface to the groundwater or refusal). The soil core lengths ranged from the surface to 107 cm (42 in), 91 cm (36 in), 91 cm (36 in), and 122 cm (48 in) below the ground surface (bgs) at locations A, B, C, and D, respectively. A 118-mL (4 oz) glass jar, two 250-mL (8 oz) high-density polyethylene (HDPE) bottles, and a 118-mL (4 oz) HDPE bottle (for moisture determination) were filled with samples, after brief mixing, at each sampling location. Loss of volatile PFAS may have occurred during sample mixing, leading to a potential low bias in the results. Therefore, the soil PFAS concentrations should be considered as minimum concentrations. A duplicate field soil sample was also collected. The 118-mL HDPE bottles were shipped to a commercial laboratory (Laboratory 1), while the 250-mL bottles were shipped to a USEPA research laboratory (Laboratory 2).

Soil gas samples were collected below the building slab or pavement and at multiple depths between the soil surface and groundwater. The general subslab and soil gas sampling systems are shown in Fig. S2. Subslab soil gas sampling ports were installed within the building by drilling a shallow 4 cm (1.5 in) hole followed by a 1.6 cm (5/8 in)-diameter hole through the remaining foundation. The subslab soil gas probes consisted of a stainless-steel Vapor Pin^®^ with a silicone sleeve [52]. Once the Vapor Pin^®^ was secured, the top barb was covered with a silicone cap completely seal between the subsurface and indoor air environment. The boreholes were covered with flush stainless-steel caps. Vapor Pins^®^ installed inside the building were leak-checked using the water dam leak check method [53]. No leaks were detected.

Multilevel exterior soil gas probes were installed at the sampling locations (Fig. S1) in clusters with a diameter of 30 cm (12 in). The exterior soil gas probes consisted of an AMS (AMS, American Falls, ID) stainless-steel dedicated tip with a stainless-steel #50 screen and silicone tubing on the surface. The soil gas probes were installed at the desired depths, with roughly equal spacing from the ground surface to the groundwater, using a slide hammer and hollow drive rods with 1.6 cm (0.625 in) outer diameter. At locations A and B, the sampling depths were 30–46 cm (12–18 in) and 61–76 cm (24–30 in) below the ground surface (bgs), respectively. At location C, the sample depths were 15–30 cm (6–12 in) and 46–61 cm (18–24 in) bgs, whereas at location D, samples were collected at 15–30 cm (6–12 in), 61–76 cm (24–30 in), and 91–107 cm (36–42 in) bgs. At locations B and C, the ground surface was covered by pavement or concrete, and the shallowest probe was installed at subslab level using a stainless-steel Vapor Pin^®^. Each Vapor Pin^®^ was leak-checked using the helium shroud tracer method [20]. No leakage was observed.

The sample volumes were guided by the total soil gas photoionization detector (PID) readings obtained during vapor probe purging. Two sample volumes were collected from each exterior and subslab soil gas sample location: (1) a 100-mL sample and (2) varying sample volumes based on the PID readings (at < 2 ppm, 1-L sample was collected; between 2 and 20 ppm, the second 100-mL sample was collected; at > 20 ppm, 10-mL sample was collected). Sample volumes of 100 mL or less were collected using a 60-mL gas-tight syringe, whereas 1-L samples were collected using a vacuum pump. The sampling flow rate was maintained at < 200 mL/min. The soil gas samples were collected by attaching silicone tubing from the sampling port to the front end of a clean multibed perfluorochemical thermal sorbent tube (Fig. S2). Thaxton et al., [40]. The syringe or pump was attached to the back end of the thermal sorbent tube, and the required soil gas volume was pulled through the sorbent tube. Once sampling was completed, the thermal sorbent tube was immediately sealed with gas-tight brass Swagelock^®^ caps. One field blank (100 mL) was collected from the ambient air. All samples were shipped on ice to Laboratory 1 and stored for ≤ 22 days prior to analysis.

Groundwater samples were collected from temporary wells installed at depths of 137–152 cm (54–60 in) bgs, using a hand auger at locations A and D. Once the water table was reached, the probes were pushed approximately 1 ft deeper. The borehole was filled with sand and sealed with hydrated granular bentonite. At location B, two attempts were made to reach the water table; however, refusal occurred at 122 cm (48 in) bgs. At location C, four attempts were made to reach the water table; however, refusal occurred at 91 cm (36 in) bgs.

The groundwater was allowed to stabilize for approximately 24 h prior to sample collection. A low-flow peristaltic pump at a rate not greater than the well recharge rate was used to collect groundwater samples into two 250-mL HDPE bottles preserved with Trizma^®^ for non-FTOH PFAS analysis and a 1-L unpreserved HDPE bottle for FTOH analysis.

### 2.3. Sample extraction and analysis

Table S2 lists the target PFAS for this investigation, including their chemical properties. In addition to FTOHs, other potentially volatile PFAS, defined by their vapor pressures (VPs) and/or HLCs, were included. The analytical standards, both the native and mass-labeled PFAS extraction standards, were purchased from Wellington Laboratories (Guelph, Ontario, Canada). Extraction chemicals for solution preparation and analytical solvents were obtained at highest purity (i.e., Optima grade) from Fisher Scientific (Waltham, Massachusetts).

The PFAS compounds in the soil samples (10 g) were extracted at Laboratory 1 using 1:1 hexane:acetone in a CEM Corporation MARS^™^ Xpress microwave [45]. Soil samples were analyzed using gas chromatography (GC)/tandem mass spectrometry/mass spectrometry (MS/MS) following Method 8270E [49], which was modified to include FTOHs, on a Thermo Scientific 1310 GC and TSQ-8000 tandem mass spectrometer. Other PFAS were analyzed by solid-phase extraction (SPE) and liquid chromatography (LC)/MS/MS following Method 537 [46] using an Exion LC system and an AB Sciex Triple Quadrupole instrument. In brief, a 250-mL water sample was fortified with surrogates and passed through a polystyrene divinylbenzene SPE cartridge. The compounds were eluted from the solid phase, and the extract concentrated to approximately 200–300 μL with nitrogen in a heated water bath. The extract was reconstituted in 1 mL with 96:4% (v/v) methanol:water, followed by the addition of the internal standards. The reporting limits (RLs) for the PFCAs and FTOHs were 0.67 and 20 ng/g, respectively.

At Laboratory 2, the soil samples were analyzed for FTOHs after the addition of surrogates and extraction in methyl tert-butyl ether (MTBE), followed by GC/MS quantitation [14,19]. In brief, the samples were analyzed using an Agilent Technologies 6890N gas chromatograph equipped with a 5973N mass-selective detector operated in positive chemical ionization mode. A Restek (Bellefonte, PA) RTX-1701 (40 m × 0.25 mm i.d., 0.25 µm film thickness) column was used for chromatographic separations. Sample volumes of 1 μL were injected into a 4-mm I.D. gooseneck inlet liner, in the pulse-splitless mode, at 40 psi for 0.90 s. The inlet and MS interface temperatures were set at 140 °C and 290 °C, respectively. The column temperature was programmed as follows: held at 60 °C for 1 min, ramped up at 3 °C/min to 75 °C, ramped up at 20 °C/min to 185 °C, followed by ballistic heating to a final temperature of 260 °C and held for 6 min. Helium was used as a carrier gas at a constant flow rate of 1 mL/min. The detection limits were defined as the concentration (ng/mL) equivalent to three times the standard deviation of replicate instrumental measurements of the lowest concentration standard of the target analyte in MTBE. The RLs at Laboratory 2 ranged from 0.13 to 0.26 ng/g; the RLs increasing as the carbon chain length increased. Only the soil samples were analyzed at Laboratory 2.

Groundwater samples were analyzed at Laboratory 1 for FTOH using GC/MS/MS in electron impact ionization mode following a modified USEPA Method 8270E [49], with an additional MS detector. The analysis of other PFAS in groundwater was performed using LC/MS/MS operated in the negative electrospray ionization mode following a modified USEPA method 537 [46]. In brief, a 250-mL groundwater sample was fortified with isotopically labeled extraction standards and passed through an SPE cartridge. The compounds were eluted from the solid phase using a proprietary combination of solvents. The extract was concentrated to approximately 200–300 μL with nitrogen in a heated water bath, and then reconstituted to 1 mL. All analytical instruments and sample handling instruments were free of Teflon^™^ and other PFAS sources.

Soil gas and subslab soil gas samples were analyzed by thermal desorption/GC/MS following Method TO-17 [44], which was modified to include tandem MS. Sample tubes and filters were thermally desorbed using a Gerstel TD 3.5+ an Agilent Technologies 8890 gas chromatograph configured with a Gerstel Cooled Injection System focusing trap. The system was equipped with an Agilent 7000D triple quadrupole mass spectrometer operated in electron ionization mode and multiple reaction monitoring (MRM) for targeted analysis. The tubes and filters were desorbed at 300 °C for 5 min in the splitless mode with transfer to a quartz wool focusing trap at − 130 °C. The trap was heated to 275 °C with a hold time of 4 min at a split flow rate of 5 mL/min (low--concentration configuration) or 200 mL/min (high-concentration configuration). The GC oven was equipped with an Agilent DB-624 UI column (60 m x 0.25 mm × 1.4 µm). The oven heating program started at 40 °C for 3 min, followed by a 15 °C/min ramp to a final temperature of 230 °C, which was held for 4.34 min. Helium was used as a carrier gas at a constant flow rate of 1 mL/min. The MS source and transfer line temperatures were maintained at 250 °C. RLs were 0.10 ng using the low-concentration 5 mL/min split-flow configuration or 5.0 ng using the high-concentration 200 mL/min split-flow configuration.

The method met all the TO-17 [44] calibration requirements for PFCA compounds using the National Institute of Standards and Technology (NIST) traceable standard solutions introduced through the thermal desorption system and characteristic MRM transitions based on precursor mass ions identified in the PFCA full scan spectra. However, subsequent detailed studies comparing the direct injection of PFOA standards into the GC inlet with thermally desorbed PFOA standards from a sorbent tube suggested that PFOA was degraded during the thermal desorption sample introduction step. The primary peak resulting from the thermal desorption of the PFOA standard had a spectrum consistent with that of perfluoro-1-heptene (PFHP), suggesting that inadvertent derivatization occurred. Therefore, the identification of PFCA is subject to potential positive interference from their corresponding perfluoroalkenes. Studies investigating thermal treatment of PFAS-impacted soils and spent water purification filters reported PFHP as a primary breakdown product of PFOA at temperatures 200–400 °C as well as other perfluorinated products, specifically perfluoroalkenes, supporting our supposition [5,57].

### 2.4. Quality control

Quality control (QC) was monitored using a 5-point minimum initial calibration, continuous calibration verification, internal standards, method blanks, field blanks, laboratory control samples, laboratory control duplicates, field duplicates, surrogate standards, matrix spikes, and matrix spike duplicates, as applicable to the reference method and matrix. The QC acceptance criteria are presented in Table S3. The blanks in most of the matrices did not contain detectable PFAS. During the soil gas and subslab soil gas analyses, slight random exceedances of the acceptance limits for the laboratory control sample/laboratory control duplicates (LCS/LCD) and continuous calibration verification samples (CCVs) were identified; however, these were within 2% of the acceptance limit. Exceedances of the upper end of the calibration range were identified for perfluorohexanoic acid (PFHxA), 6:2 FTOH, and 8:2 FTOH, resulting in the “E” data flag for the subslab soil gas samples. The groundwater QC measures were generally within the acceptance limits. Exceedance of surrogate spike recovery, low matrix spike recovery of PFBS by 5–15%, and LCS/LCD precision values slightly outside the acceptance limits were identified in the groundwater data. Soil QC measures indicate a potentially low bias due to the LCS and matrix spike recoveries within 11% of the acceptance limits. The QC measures outages were randomly distributed throughout the datasets, varying by matrix, sample, and compound. The overall data were acceptable, and deviations did not have a major effect on the interpretation of the results. Although volatile losses may have occurred during soil sampling, the presence of the remaining PFAS was sufficient to show their relative concentrations and distributions (assuming that losses were equivalent among samples that underwent the same sample-handling procedures).

### 2.5. Soil characterization

The field descriptive notes of the soil cores collected at sites A–D were recorded during sampling (Table S1). For visual characterization, the soil columns were segmented into layers based on texture, color, and redox features (i.e., mottling). Each soil core was analyzed for the following parameters: particle size distribution using the hydrometer method, pH in a 1:1 soil:0.01 M CaCl_2_ suspension, and total organic carbon by combustion at 1350^◦^ F and infrared detection [27].

## 3. Results

### 3.1. Subslab and soil gas

In general, the soil gas FTOH concentrations were higher at the subslab locations (SG01 and SG02) than nearly all other locations and depths; however, they decreased as the distance from the presumed source building increased (Table 1). The subslab soil gas concentrations ranged from 160 to 12,000 E µg/m^3^ for individual FTOHs. 4:2 FTOH was detected only in the subslab soil gas sample beneath the presumed source building. At location C, which was approximately 43 m (140 ft) away from the presumed source building, the concentrations of 5:2 and 7:2 secondary FTOH (sFTOH) increased as the depth from the surface increased. The peak concentrations of 5:2 (590 µg/m^3^) and 7:2 sFTOHs (160 µg/m^3^) were recorded at 61 cm (24 in) bgs. The concentrations of 6:2, 8:2, and 10:2 FTOHs at location C were the highest immediately under the pavement (i.e., subslab), slightly elevated (7–18%) at 61 cm (24 in) bgs, and the lowest at 30 cm (12 in) bgs. At location B, approximately 76 m (250 ft) east-southeast of the presumed source building, the concentrations of 6:2, 8:2, and 10:2 FTOHs ranged from 2.2 to 14 µg/m^3^; however, concentrations of 4:2, 5:2s, and 7:2 sFTOHs were not detected. At locations A and D, the furthest sampling locations from the presumed source building (104 and 84 m (340 and 275 ft), respectively, away from the source building), only 5:2 sFTOH at 30 cm (12 in) bgs at location A and 6:2 FTOH at 46 cm (18 in) bgs at location D were detected.

Three PFCA compounds—perfluorobutanoic acid (PFBA), PFHxA, and PFOA—were detected in the subslab soil gas sample directly beneath the presumed source building at concentrations ranging from 59 to 650 E µg/m^3^ (Table 1). The three PFCAs as well as perfluoropentanoic acid (PFPeA) and perfluoroheptanoic acid (PFHpA) were detected at location C at concentrations ranging from 0.7 to 180 µg/m^3^. Only PFBA was marginally detected at location D; and PFCAs were not at locations A and B.

### 3.2. Soil

All the target PFCAs were identified in the soil samples at locations A, B, and C (Table 2). The soil samples at location D had PFCA chain lengths ranging from C4 to C10. The anticipated spatial pattern of PFCAs was observed in the soil samples at location C, which had higher concentrations than the samples collected from other locations further away from the presumed source. The PFCA concentrations in the soil samples at location C ranged from 4.3 to 810 ng/g while those at locations B, D (when detected), and A, ranged from 0 to 220, 0–53, and 0–30 ng/g, respectively.

FTOHs were not detected in the soil samples analyzed at Laboratory 1; however, FTOHs at concentrations lower than the RLs of FTOHs at Laboratory 1 were identified at Laboratory 2 (Table 3). The RLs were 20 ng/g for the FTOHs identified at Laboratory 1 and 0.13–0.26 ng/g at Laboratory 2. The most abundant soil FTOH was 10:2 FTOH with concentrations ranging from < 0.26–36.15 ng/g, followed by 8:2 and 6:2 FTOHs with concentrations > 1 ng/g at nearly all sampling locations. The sFTOHs were found primarily at location C, with concentrations between 4.5 and 5.0 ng/g. A small quantity (0.15 ng/g) of 5:2 sFTOH was found at location A.

Higher PFAS concentrations can be related to the greater sorptive ability associated with a higher total organic carbon content (TOC) (Table S1) at location C [18,23]. In contrast, the soil samples from location C also had the lowest clay content among all the soil samples from all sampling locations. Loganathan and Wilson [29] found that as the kaolinite clay content increased, the sorption of PFAS increased, irrespective of the functional group. While the clay mineralogy at the test site is unknown, the marked contrast in TOC content (approximately 8% versus approximately 2%) among the sampling locations may be a controlling factor for PFAS sorption at location C.

### 3.3. Groundwater

FTOHs were not detected in groundwater samples at any sampling location (Table 2). PFCAs with carbon chain lengths ranging from perfluorobutanoic acid (C4) to perfluoroundecanoic acid (C11) were detected. PFHxA had the highest PFCA concentration (37–49 µg/L). Groundwater concentrations for shorter chain PFCAs (≤ 10 C) ranged from 1.2 to 49 µg/L and were similar in concentration to the soil results in ng/g. In contrast, higher soil PFCA concentrations were observed compared to groundwater for the longer chain (≥ 11 C) compounds, which fits with a general reported pattern of lower aqueous solubility for the longerchain PFCAs [22]. The anticipated spatial pattern of higher PFCA concentrations near the presumed source building than at locations further away was generally observed, with concentrations being higher in samples from location D than those from location A.

## 4. Discussion

### 4.1. PFAS distribution

PFAS concentrations in the soil gas phase were the highest (when detected) in the subslab soil gas and tended to decrease as the distance from the presumed source building increased, as evidenced by the absence of PFAS at locations A and D. The absence of FTOHs in the groundwater regardless of the distance from the presumed source building (Table 2) and their presence in the soil and soil gas indicate that FTOHs likely migrated in the vapor phase. The FTOH distribution within the soil column where the highest concentrations were found in the subslab soil gas followed by those collected from the deepest sampling depth, support this concept. The driving force for this vapor-phase transport can be attributed to diffusion and advection caused by barometric pumping from superficial openings during weather events [41]. Advective driving forces can exist in the vadose zone because of the pressure gradients around the site buildings (Fig. S1) [47], leading to FTOH transport from the source to the distant sampling locations. Alternatively, if there were cracks, openings in the concrete/asphalt covering the ground surface, or grass- or gravel-covered open areas between the presumed source building and sampling locations, atmospheric deposition of PFAS may have occurred. The PFAS deposited on the ground surface may be washed through the openings into the subsurface during rainfall or snow melting [23].

In contrast to FTOHs, the presence of sFTOHs at greater concentrations at deeper sampling sites could be the result of the concomitant vertical diffusion/dispersion of the FTOHs into the underlying soil through cycles of reduction and oxidation to produce sFTOHs following the transformation pathways described by Evich et al. [15]. Changing redox conditions in the subsurface are indicated in the field notes (Table S1) by the presence of “redox features.” If sFTOHs were generated at relatively high concentrations, such as at location C, they could diffuse or advect towards the surface into the oxidizing coarse sand and cobble layer found under the slab, degrade further, and contribute to the vapor-phase PFCA concentrations.

PFCAs were found in all measured phases and locations, with concentrations generally decreasing from underneath the presumed source building with increasing lateral distance from the building. Within the soil column, the PFCA concentrations were higher at deeper locations and decreased towards the surface. This distribution indicates groundwater transport from the presumed source to the sampling locations. Additionally, chemical transformations of PFAS in the subsurface may have occurred, leading to increased concentrations of PFCAs at depths in the soil and groundwater [7]. At locations A and D, the PFCA concentrations were greater in the groundwater than in the soil for compounds C4–C7. In contrast, C8–C10 PFCAs were more abundant in soil than in groundwater. No soil gas-phase PFCAs were identified at either location. In addition to chemical transformations, greater soil sorption of C8 and longer PFCA may have occurred during normal cycles of water table rise and fall, leading to higher soil concentrations at greater depths.

Although the presence of PFAS at the site was expected because of the long production history of fluoroelastomers and fluorotelomers, the presence of PFCAs (PFBA, PFHxA, PFHpA, and PFOA) in the soil gas phase was unexpected because of their very low volatility. PFCAs are generally considered to be predominantly partitioned into the particulate phase in the atmosphere; however, reports of PFBA, PFHpA, PFHxA, PFOA, and PFPeA in the gas phase at ambient air sites at concentrations below 0.025 ng/m^3^ exist in the literature [26,39,6]. Gas-phase PFHpA, PFHxA, and PFOA at concentrations below 2.5 ng/m^3^ have been reported in indoor air at background sites [6]. Kaiser et al. [25] were able to measure sublimation of PFOA at 45 °C and also measured some partitioning into the air phase from an aqueous solution at pH 7. Zhang et al. [60] reported the sublimation vapor pressures for FTOH and PFCA using a method that involved observing the mass loss under vacuum at near-ambient temperatures.

At location C, where nearly all the PFAS were found, comparing soil gas concentrations expected at equilibrium with the observed PFCA soil concentrations, the calculated soil gas concentrations were generally lower than the observed equilibrium soil gas concentrations (Table S4). One exception to this trend was PFOA where the observed soil gas concentrations at all 3 sampled depths were within the lower 5% range of calculated equilibrium concentrations (5.89 E-4-232 µg/m^3^). The soil gas concentrations of 4:2, 6:2, 8:2, and 10:2 FTOH were predicted to be approximately two orders of magnitude higher than the observed concentrations.

Higher soil gas concentrations of PFCAs than expected, based on equilibrium calculations at location C (Table S4), led to the possibility that a mist or vapor phase was present. This result is attributable to the assumption in the equilibrium calculation that PFCAs are partitioned into soil pore space water or water associated with mineral and/or organic particles. When partitioned, PFCAs are ionized at the observed circumneutral pH values, and the ionized forms do not volatilize. However, Bastow et al. [9] argued that when organic solvents are present (including fuels or asphalt), PFAS have very different pKa values and are thus less likely to be ionized in their presence. The sampling locations are known to have other volatile and semi-volatile chemical effects, including organic solvents. Alternatively, the degradation of FTOHs to PFCAs may have occurred in the vadose zone [3,15].

## 5. Conclusions

The PFAS with the highest concentrations at the test sites were 6:2 FTOH, 8:2 FTOH, 10:2 FTOH, 5:2 sFTOH, and PFHxA. FTOH concentrations in the soil gas were generally more prevalent in the subslab soil gas underneath the presumed source building and tended to decrease with increasing distance from the presumed source building. However, the sFTOH concentrations were higher at greater depths toward the groundwater.

The FTOH concentrations in the soil samples were low (<37 ng/g). The higher PFCA concentrations at deeper sampling points toward the groundwater indicate that the migration in and release of PFCAs from the groundwater source are indicated.

FTOHs were not detected in the groundwater at any sampling location; however, PFCAs with carbon chains from C4 to C10 were detected in all the collected samples.

PFAS concentrations in soil and groundwater exceeded state and federal thresholds, where established, at all sampled locations. With a source of PFAS in the soil and groundwater as well as its presence in the soil gas (including subslab soil gas), the potential for VI has been recognized. However, a final confirmatory analysis of indoor air to complete the exposure pathway is lacking. The VI of volatile PFAS may pose health risks to building occupants and therefore, warrants consideration during VI assessments at facilities where high PFAS concentrations are present in shallow soil and the groundwater.

### Environmental implication

Volatile per- and polyfluoroalkyl substances (PFAS) pose an inhalation exposure risk to occupants of buildings overlying a contaminated soil or groundwater source. Many PFAS compounds are volatile and semivolatile, and partition between atmospheric particulate matter, mists, and the vapor phase. PFAS were found in groundwater, soil, and soil gas at a manufacturing facility in New Jersey, USA. With PFAS presence in the vapor phase, the potential for vapor intrusion has been recognized and PFAS should; thus, be considered as a potential hazard during contaminated site assessments.

## Supplementary Material

Supplement1

## Figures and Tables

**Table 1:** Vapor concentrations in the subslab vapor, soil gas, and ambient air samples analyzed at Laboratory 1.

			Sample. SG01	Sample SG02	Sample Location A 1.5′	Sample Location A 2.5′	Sample Location B Subslab	Sample Location B 1.5′	Sample Location B 2.5′	Sample Location C Subslab	Sample Location C 1′	Sample Location C 2′	Sample Location D 1′	Sample Location D 2.5′	Sample Location D 3.5′	Blank Site (1 L sample)

Class	Chemical Name	Acronym	Vapor Concentration in Subslab Soil Gas (µg/m^3^)		Soil Gas Concentration (µg/m^3^)											Ambient Air (µg/m^3^)
Perfluorinated carboxylic acids (PFCAs)	Perfluorobutanoic acid	PFBA	140	99	< 1.0	< 1.0	< 50	< 1.0	< 1.0	0.84	7.1 E	23	1.0 J	< 1.0	< 1.0	0.12
	Perfluoropentanoic acid	PFPeA	< 50	< 50	< 1.0	< 1.0	< 50	< 1.0	< 1.0	4 I	25 E,I	62	< 1.0	< 1.0	< 1.0	< 0.10
	Perfluorohexanoic acid	PFHxA	460	650 E*	< 1.0	< 1.0	< 50	< 1.0	< 1.0	7.1 E	49 E	180	< 1.0	< 1.0	< 1.0	0.16
	Perfluoroheptanoic acid	PFHpA	< 50	< 50	< 1.0	< 1.0	< 50	< 1.0	< 1.0	< 0.10	1.3	3.8	< 1.0	< 1.0	< 1.0	< 0.10
	Perfluorooctanoic acid	PFOA	59	62	< 1.0	< 1.0	< 50	< 1.0	< 1.0	0.72	1.7	7	< 1.0	< 1.0	< 1.0	< 0.10
Perfluorinated telomere alcohols (FTOHs)	2-perfluorobutyl ethanol	4:2 FTOH	300	290	< 1.0	< 1.0	< 50	< 1.0	< 1.0	< 0.10	< 0.10	< 0.10	< 1.0	< 1.0	< 1.0	< 0.10
	1-perfluoropentyl ethanol	5:2 sFTOH	930	2800	< 1.0	< 1.0	< 50	< 1.0	< 1.0	4.9	64 E	590	1.0 J	< 1.0	< 1.0	< 0.10
	1-perfluoroheptyl ethanol	7:2 sFTOH	220	180	< 1.0	< 1.0	< 50	< 1.0	< 1.0	1.1	22 E	160	< 1.0	< 1.0	< 1.0	< 0.10
	2-perfluorohexyl ethanol	6:2 FTOH	12000 E	8800 E	2.6	< 2.0	< 50	3	3.1	85 E	0.23	1.3	< 1.0	< 2.0	< 2.0	5.5
	2-perfluorooctyl ethanol	8:2 FTOH	6100 E	1700	< 1.0	< 1.0	< 50	14	6	27 E	0.15	2.1	< 1.0	< 1.0	< 1.0	< 0.10
	2-perfluorodecyl ethanol	10:2 FTOH	930	160	< 1.0	< 1.0	< 50	2.2	< 1.0	14 E	1.3	7.7	< 1.0	< 1.0	< 1.0	< 0.10

* E: Results exceeding the calibration range. I = Matrix interference affecting the qualifier ion(s) used for compound identification. J – Estimated value due to potential carryover from previously analyzed high-concentration samples.

**Table 2:** Concentrations of polyfluoroalkyl substances in the soil and groundwater water samples analyzed at Laboratory 1.

			Sample Location A	Sample Location B	Sample Location B Dup	Sample Location C	Sample Location D	Sample Location A	Sample Location D	Sample Location D – Duplicate

Class*	Chemical Name	Acronym	Soil Concentration (ng/g)					Groundwater Concentration (μg/L)		
Perfluorinated carboxylic acids (PFCAs)	Perfluorobutanoic acid	PFBA	*<* 2.2	*<* 2.2	*<* 2.2	4.3	*<* 2.2	1.9	4.3	4.3
	Perfluoropentanoic acid	PFPeA	2	0.95	0.97	18	2.9	5.3	8.7	8.4
	Perfluorohexanoic acid	PFHxA	16	3.7	3.8	190	12	49	39	37
	Perfluoroheptanoic acid	PFHpA	1.5	*<* 0.67	*<* 0.67	71	14	1.5	4.5	4.7
	Perfluorooctanoic acid	PFOA	3.3	1.5	1.5	480	53	1.2	7.2	7.3
	Perfluorononanoic acid	PFNA	5.6	1.4	1.4	180	23	4.3	7.8	7.2
	Perfluorodecanoic acid	PFDA	30	6.1	6	810	2.2	5.2	1.8	1.8
	Perfluoroundecanoic acid	PFUnA, or PFUndA	13	8.5	13	290	*<* 0.67	*<* 0.02	*<* 0.02	*<* 0.02
	Perfluorododecanoic acid	PFDoA, or PFDoDA	27	92	120	640	*<* 0.67	0.069	*<* 0.02	*<*0.02
	Perfluorotridecanoic acid	PFTrDA, or PFTriA	9.2	50	45	140	*<* 0.67	*<* 0.02	*<* 0.02	*<* 0.02
	Perfluorotetradecanoic acid	PFTeDA, or PFTeA	24	220	140	410	*<* 0.67	*<* 0.02	*<* 0.02	*<* 0.02
	Perfluorohexadecanoic acid	PFHxDA	7.8	78	47	190	*<* 0.67	*<* 0.02	*<* 0.02	*<* 0.02
	Perfluorooctadecanoic acid	PFODA	3.1	23	17	61	*<* 0.67	*<* 0.02	*< <*0.02	*<* 0.02

* FTOHs were not detected in the soil or groundwater.

**Table 3:** Concentrations of fluorotelomer alcohols in the soil samples from the New Jersey site that were analyzed at Laboratory 2.

Chemical Identification			Sample Location A (ng/g)	Sample Location B (ng/g)	Sample Location B (ng/g) Dup	Sample Location C (ng/g)	Sample Location D (ng/g)
Chemical Class	Chemical Name	Substance Acronym					
**Perfluorinated telomer alcohols (FTOHs)**	2-perfluorobutyl ethanol	4:2 FTOH	0.05*	0.06	0.38	0.12	0.09
	1-perfluoropentyl ethanol	5:2 sFTOH	0.15	< 0.22	< 0.22	4.78	< 0.22
	1-perfluoroheptyl ethanol	7:2 sFTOH	< 0.13	< 0.13	< 0.13	4.91	< 0.13
	2-perfluorohexyl ethanol	6:2 FTOH	1.45 J^†^	3.24 J	1.71	2.38	< 0.21
	2-perfluorooctyl ethanol	8:2 FTOH	1.01 J	2.92 J	6.71 J	3.56	0.48 J
	2-perfluorodecyl ethanol	10:2 FTOH	4.48 J	20.85	3.44 J	36.15	< 0.26

* All values are the averages of replicate analyses.

† J – estimated value.

## Data Availability

Data will be made available on request.

## HIGHLIGHTS

- Per- and polyfluoroalkyl substances (PFAS) pose serious health risks.
- PFAS in soil, soil gas, and groundwater at a site in New Jersey were analyzed.
- Fluorotelomer alcohols were detected in soil and soil gas samples.
- PFCAs were found in soil, soil gas, and groundwater samples.
- PFAS are capable of vapor intrusion.

## Abbreviations:

PFAS: polyfluoroalkyl substances
AFFFs: aqueous-film-forming foams
FTOHs: fluorotelomer alcohols
HLCs: Henry’s Law constants
PFOA: perfluorooctanoic acid
PFCAs: perfluoroalkyl carboxylic acids
HDPE: high-density polyethylene
PID: photoionization detector
VPs: vapor pressures
SPE: solid-phase extraction
RLs: reporting limits
MTBE: methyl tert-butyl ether
MRM: multiple reaction monitoring
PFHP: perfluoro-1-heptene
QC: quality control
LCS/LCD: laboratory control sample/laboratory control duplicates
CCVs: continuing calibration verification samples
PFBA: perfluorobutanoic acid
PFHxA: perfluorohexanoic acid
PFPeA: perfluoropentanoic acid
PFHpA: perfluoroheptanoic acid
TOC: total organic carbon content
bgs: below ground surface

## References

[1] Abusallout I, Holton C, Wang J, Hanigan D, 2022. Henry’s law constants of 15 per- and polyfluoroalkyl substances by static headspace analysis. J Hazard Mater 3, 100070. 10.1016/j.hazl.2022.100070.

[2] Ahrens L, Shoeib M, Del Vento S, Codling G, Halsall C, 2011. Polyfluoroalkyl compounds in the Canadian arctic atmosphere. Environ Chem 8, 399–406. 10.1071/EN10131.

[3] Ahrens L, Harner T, Lee SC, Guo R, Reiner EJ, 2012. Improved characterization of gas-particle partitioning for per- and polyfluoroalkyl substances in the atmosphere using annular diffusion denuder samplers. Environ Sci Technol 46, 7199–7206. 10.1021/es300898s. 22606993

[4] Ahrens L, Harner T, Shoeib M, Koblizkova M, Reiner EJ, 2013. Characterization of two passive air samplers for per- and polyfluoroalkyl substances. Environ Sci Technol 47, 14024–14033. 10.1021/es4048945. 24219299

[5] Alinez A, Sasi PC, Zhang P, Yao B, Kubatova A, Golovko SA, , 2022. An investigation of thermal air degradation and pyrolysis of PFAS and AFFF in soil. ACS EST Eng 2, 198–209. 10.1021/acsestengg.1c00335.

[6] Assouline S, Kamai T, 2019. Liquid and vapor water in vadose zone profiles above deep aquifers in hyper-arid environments. Water Resour Res 55, 3619–3631. 10.1029/2018WR024435.

[7] Balgooyen S, Remucal CK, 2023. Impacts of environmental and engineered processes on the PFAS fingerpring of fluorotelomer-based AFFF. Environ Sci Technol 57, 244–254. 10.1021/acs.est.2c06600. 36573898

[8] Barber JL, Berger U, Chaemfa C, Huber S, Jahnke A, Temme C, , 2007. Analysis of per and polyfluorinated alkyl substances in air samples from Northwest Europe. J Environ Monit 9, 530–541. 10.1039/B701417A. 17554424

[9] Bastow TP, Douglas GB, Davis GB, 2022. Volatilization potential of per-and poly-fluoroalkyl substances from airfield pavements and during recycling of asphalt. Environ Toxicol Chem 41, 2202–2208. 10.1002/etc.5425. 35781701 PMC9540562

[10] Bertin H, Del Campo Estrada E, Atteia O, 2017. Foam placement for soil remediation. Environ Chem 14, 338–343. 10.1071/EN17003.

[11] Bokkers GGH, van den Ven B, Janssen P, Bil W, van Broekhuizen F, Zeilmaker M, , 2019. Per- and polyfluoroalkyl substances (PFAS) in food contact materials. Institute for Public Health and the Environment (RIVM) Bilthoven (NL),, National, p. 112.

[12] Del Vento S, Halsall C, Gioia R, Jones K, Dachs J, 2012. Volatile per– and polyfluoroalkyl compounds in the remote atmosphere of the western Antarctic Peninsula: an indirect source of perfluoroalkyl acids to Antarctic waters? Atmos Pollut Res 3, 450–455. 10.5094/APR.2012.051.

[13] Environmental Council of the States (ECOS). 2023. Processes & considerations for setting State PFAS standards Washington DC. https://www.ecos.org/wp-content/uploads/2023/03/2023-ECOS-PFAS-Standards-Paper-Update.pdf (accessed 13, November 2023).

[14] Ellington JJ, Washington JW, Evans JJ, Jenkins TM, Hafner SC, Neill MP, 2009. Analysis of fluorotelomer alcohols in soils: optimization of extraction and chromatography. J Chromatogr A 1216, 5347–5354. 10.1016/j.chroma.2009.05.035. 19497578

[15] Evich MG, Davis MJB, McCord JP, Acrey B, Awkerman JA, Knappe DRU, , 2022. Per- and polyfluoroalkyl substances in the environment. Science 375 (6580). 10.1126/science.abg9065. PMC8902460 35113710

[16] Hamid H, Li LY, Grace JR, 2018. Review of the fate and transformation of per- and polyfluoroalkyl substances (PFASs) in landfills. Environ Pollut 235, 74–84. 10.1016/j.envpol.2017.12.030. 29275271

[17] Hammer J, Endo S, 2022. Volatility and nonspecific van der Waals interaction properties of per- and polyfluoroalkyl substances (PFAS): evaluation using hexadecane/air partition coefficients. Environ Sci Technol 56, 15737–15745. 10.1021/acs.est.2c05804. 36240042 PMC9671037

[18] He A, Liang Y, Li F, Lu Y, Liu C, Li J, , 2022. Vital environmental sources for multitudinous fluorinated chemicals: new evidence from industrial byproducts in multienvironmental matrices in a fluorochemical manufactory. Environ Sci Technol 56, 16789–16800. 10.1021/acs.est.2c04372. 36354080

[19] Henderson WM, Weber EJ, Duirk SE, Washington JW, Smith MA, 2007. Quantification of fluorotelomer-based chemicals in mammalian matrices by monitoring perfluoroalkyl chain fragments with GC/MS. J Chromatogr B 846, 155–161. 10.1016/j.jchromb.2006.08.042. 17000139

[20] Interstate Technology & Regulatory Council (ITRC). 2007. Vapor Intrusion Pathway: A Practical Guideline. VI-1. Washington, D.C.: Interstate Technology & Regulatory Council, Vapor Intrusion Team 〈https://itrcweb.org/teams/projects/vapor-intrusion〉. (accessed 18 September 2023).

[21] Interstate Technology & Regulatory Council. 2023a. PFAS Technical and Regulatory Guidance Document (August 2023) – PFAS Water and Soils Value Table Excel file Washington DC. https://pfas-1.itrcweb.org/#1_3 (accessed 13 November 2023).

[22] Interstate Technology & Regulatory Council. 2023b. PFAS Technical and Regulatory Guidance Document (September 2023) Washington DC. https://pfas-1.itrcweb.org/#1_7 (accessed 13 November 2023).

[23] Jia X, Li X, Zhou L, Hui Y, Li W, Cai Y, , 2023. Variations of the level, profile, and distribution of PFAS around POSF manufacturing facilites in China: an overlooked source of PFCA. Environ Sci Technol 57, 5264–5274. 10.1021/acs.est.2c08995. 36939348

[24] Johansson JH, Salter ME, Acosta Navarro JC, Leck C, Nilsson ED, Cousins IT, 2019. Global transport of perfluoroalkyl acids via sea spray aerosol. Environ Sci: Process Impacts 21635–21649. 10.1039/C8EM00525G. 30888351

[25] Kaiser MA, Dawson BJ, Barton CA, Botelho MA, 2010. Understanding potential exposure sources of perfluorinated carboxylic acids in the workplace. Ann Occup Hyg 54, 915–922. 10.1093/annhyg/meq066. 20974675 PMC2984390

[26] Kim SK, Kannan K, 2007. Perfluorinated acids in air, rain, snow, surface runoff, and lakes: relative importance of pathways to contamination of urban lakes. Environ Sci, Technol 41, 8328–8334. 10.1021/es072107t. 18200859

[27] Kissel DE, Sonon LS, 2011. Soil test handbood for Georgia. Univerity of Georgia Cooperative Extension Service Special Bulletin 62. 〈https://secure.caes.uga.edu/extension/publications/files/pdf/SB%2062_2.PDF〉. (accessed 18 September 2023).

[28] Lei YD, Wania F, Mathers D, Mabury SA, 2004. Determination of vapor pressures, octanol—air, and water—air partition coefficients for polyfluorinated sulfonamide, sulfonamidoethanols, and telomer alcohols. J Chem Eng Data 49, 1013–1022. 10.1021/je049949h.

[29] Loganathan N, Wilson AK, 2022. Adsorption, structure, and dynamics of short- and long-chain PFAS molecules in kaolinite: Molecular-level insights. Environ Sci Technol 56, 8043–8052. 10.1021/acs.est.2c01054. 35543620

[30] Maire J, Fatin-Rouge N, 2017. Surfactant foam flushing for in situ removal of DNAPLs in shallow soils. J Hazard Mater 321, 247–255. 10.1016/j.jhazmat.2016.09.017. 27631687

[31] Masoner JR, Kolpin DW, Cozzarelli IM, Smalling KL, Bolyard SC, Field JA, , 2020. Landfill leachate contributes per-/poly-fluoroalkyl substances (PFAS) and pharmaceuticals to municipal wastewater. Environ Sci Water Res Technol 6, 1300–1311. 10.1039/D0EW00045K.

[32] Morales-McDevitt ME, Becanova J, Blum A, Bruton TA, Vojta S, Woodward M, , 2021. The air that we breathe: Neutral and volatile PFAS in indoor air. Environ Sci Technol Lett 8, 897–902 10.1021/acs.estlett.1c00481. 35359817 PMC8963212

[33] National Academies of Sciences, Engineering, and Medicine and National Academy of Engineering, 2022. Indoor Exposure to Fine Particulate Matter and Practical Mitigation Approaches: Proceedings of a Workshop The National Academies Press, Washington, DC. 10.17226/26331. 36067346

[34] New Jersey Department of Environmental Protection (NJ DEP). 2021. N.J.A.C. 7: 26D: Remediation Standards. Appendix 10: Chemical and physical properties of contaminants https://dep.nj.gov/wp-content/uploads/rules/rules/njac7_26d.pdf (accessed 13 November 2023).

[35] Peng-Fei Y, Dong S, Manz KE, Liu C, Woodcock MJ, Mezzari MP, , 2022. Biotransformation of 8:2 fluorotelomer alcohol in soil from aqueous film-forming foams (AFFFs)-impacted sites under nitrate-, sulfate-, and iron-reducing conditions. Environ Sci Technol 56, 13728–13739. ⟨ 10.1021/acs.est.2c03669⟩. 36127292

[36] Place BJ, Field JA, 2012. Identification of novel fluorochemicals in aqueous film-forming foams used by the US military. Environ Sci Technol 46, 7120–7127. 10.1021/es301465n. 22681548 PMC3390017

[37] Ruan T, Wang Y, Zhang Q, Ding L, Wang P, Qu G, , 2010. Trace determination of airborne polyfluorinated iodine alkanes using multisorbent thermal desorption/gas chromatography/high resolution mass spectrometry. J Chromatogr A 1217, 4439–4447. 10.1016/j.chroma.2010.04.051. 20483422

[38] Sha B, Dahlberg A-KD, Wiberg K, Ahrens L, 2018. Fluorotelomer alcohols (FTOHs), brominated flame retardants (BFRs), organophosphorus flame retardants (OPFRs) and cyclic volatile methylsiloxanes (cVMSs) in indoor air from occupational and home environments. Environ Pollut 241, 319–330. 10.1016/j.envpol.2018.04.032. 29843014

[39] Shoeib M, Harner T, Webster GM, Lee SC, 2011. Indoor sources of poly- and perfluorinated compounds (PFCS) in Vancouver, Canada: implications for human exposure. Environ Sci Technol 45, 7999–8005. 10.1021/es103562v. 21332198

[40] Thaxton K, Stuff J, Whitecavage JA, Hayes H, Miller J, 2020. Analysis of PFAS compounds in air using solid sorbent tubes with thermal desorption gas chromatography mass spectrometry. Presented at National Environmental Monitoring Conference https://apps.nelac-institute.org/nemc/2020/docs/presentations/pdf/8-4-20-Air%20Methods,%20Monitoring,%20and%20Technology-3.03-Thaxton.pdf. (accessed 18 September 2023).

[41] Tillman FD Jr, Smith JA, 2005. Site characteristics controlling airflow in the shallow unsaturated zone in response to atmospheric pressure changes. Environ Eng Sci 22, 25–37. 10.1089/ees.2005.22.25.

[42] Titaley IA 2021. Volatile Per- and Polyfluoroalkyl Substances (PFAS) Analysis in Landfill Gas Using Thermal Desorption Coupled With Gas Chromatography Mass Spectrometry” Presented at SETAC North America 42nd Annual Meeting

[43] Titaley I, Khattak J, Dong J, Olivares CI, DiGuiseppi B, Lutes CC, , 2022. Neutral per- and polyfluoroalkyl substances, butyl carbitol, and organic corrosion inhibitors in aqueous film-forming foams: implications for vapor intrusion and the environment. Environ Sci Technol 56, 10785–10797. 10.1021/acs.est.2c02349. 35852516

[44] USEPA. 1999. Compendium of Methods for the Determination of Toxic Organic Compounds in Ambient Air Second Edition, Compendium Method TO-17 Determination of Volatile Organic Compounds in Ambient Air Using Active Sampling Onto Sorbent Tubes EPA/625/R-96/010b.

[45] USEPA. 2007. Method 3546 Microwave Extraction February. ⟨https://www.epa.gov/sites/default/files/2015-12/documents/3546.pdf⟩. (accessed 18 September 2023).

[46] USEPA. 2009. Method 537. Determination Of Selected Perfluorinated Alkyl Acids In Drinking Water By Solid Phase Extraction And Liquid Chromatography/Tandem Mass Spectrometry (LC/MS/MS), Version 1.1 ⟨https://cfpub.epa.gov/si/si_public_record_report.cfm?Lab=NERL&dirEntryId=198984&simpleSearch=1&searchAll=EPA%2F600%2FR-08%2F092⟩. (acccessed 18 September 2023).

[47] USEPA. 2012. Conceptual model scenarios for the vapor intrusion pathway ⟨https://www.epa.gov/sites/default/files/2015-09/documents/vi-cms-v11final-2-24-2012.pdf⟩. (accessed 18 September 2023).

[48] USEPA. 2015. OSWER technical guide for assessing and mitigating the vapor intrusion pathway from subsurface vapor sources to indoor air. Office of Solid Waste and Emergency Response (OSWER), OSWER Publication 9200.2–154. ⟨https://www.epa.gov/sites/production/files/2015-09/documents/oswer-vapor-intrusion-technical-guide-final.pdf⟩. (accessed 18 September 2023).

[49] USEPA. 2018. EPA Method 8270E (SW-846): Semivolatile Organic Compounds by Gas Chromatography/Mass Spectrometry (GC-MS) https://www.epa.gov/sites/default/files/2020-10/documents/method_8270e_update_vi_06-2018_0.pdf. (accessed 18 September 2023).

[50] USEPA. 2022a. Our Current Understanding of the Human Health and Environmental Risks of PFAS ⟨https://www.epa.gov/pfas/our-current-understanding-human-health-and-environmental-risks-pfas⟩. (accessed 18 September 2023).

[51] USEPA. 2022b. EPA’s PFAS Strategic Roadmap: A Year of Progress. EPA-800-K-22-001 ⟨https://www.epa.gov/system/files/documents/2022-11/PFAS%20Roadmap%20Progress%20Report_final_Nov%2017.pdf⟩. (accessed 18 September 2023).

[52] Vapor Pin Enterprises, Inc. 2021a. Standard operating procedure: Installation and extraction of the Vapor Pin^®^ sampling device. https://www.vaporpin.com/wp-content/uploads/2021/02/Vapor-Pin-Installation-and-Extraction-SOP-3-16-2018-Web-02022021.pdf. (accessed 18 September 2023).

[53] Vapor Pin Enterprises, Inc. 2021b. Standard Operating Procedure Leak Testing the VAPOR PIN^®^ Sampling Device Via Water Dam. https://www.vaporpin.com/wp-content/uploads/2021/02/VP-Leak-Testing-with-Water-Dam-SOP-02022021.pdf. (accessed 18 September 2023).

[54] Wang Z, DeWitt JC, Higgins CP, Cousins IT, 2017. A never-ending story of per- and polyfluoroalkyl substances (PFASs)? Environ Sci Technol 51, 2508–2518. 28224793 10.1021/acs.est.6b04806

[55] Wang N, Szostek B, Buck RC, Folsom PW, Sulecki LM, Gannon JT, 2009. 8–2 Fluorotelomer alcohol aerobic soil biodegradation: pathways, metabolites, and metabolite yields. Chemosphere 75, 1089–1096. 10.1016/j.chemosphere.2009.01.033. 19217141

[56] Wu Y, Chang WC, 2012. Development of analysis of volatile polyfluorinated alkyl substances in indoor air using thermal desorption-gas chromatography-mass spectrometry. J Chromatogr A 1238, 4439–4447. 10.1016/j. chroma.2012.03.053. 22494639

[57] Xiao F, Sasi PC, Yao B, Kubatova A, Golovko SA, Golovko MY, , 2020. Thermal stability and decomposition of perfluoroalkyl substances on spent activated carbon. Environ Sci Technol Lett 7, 343–350. 10.1021/acs.estlett.0c00114.

[58] Yamazaki E, Taniyasu S, Wang X, Yamashita N, 2021. Per- and polyfluoroalkyl substances in surface water, gas and particle in open ocean and coastal environment. ISSN 0045–6535 Chemosphere 272, 129869. 10.1016/j.chemosphere.2021.129869. 33592511

[59] Yoo H, Washington JW, Ellington JJ, Jenkins TM, Neill MP, 2010. Concentrations, distribution, and persistence of fluorotelomer alcohols in sludge-applied soils near Decatur, Alabama, USA. Environ Sci Technol 44, 8397–8402. 10.1021/es100390r. 20949952

[60] Zhang M, Yamada K, Bourguet S, Guelfo J, Suuberg EM, 2020. Vapor pressure of nine perfluoroalkyl substances (PFASs) determined using the Knudsen Effusion Method. J Chem Eng Data 65, 2332–2342 10.1021/acs.jced.9b00922. 32968326 PMC7505237

