# Supplementary material for: Distribution of select per- and polyfluoroalkyl substances at a chemical manufacturing plant
Source: J Hazard Mater. Author manuscript; Available in PMC 2025 Feb 15. (PMC10734402; doi:10.1016/j.jhazmat.2023.133025)
Supplement: Supplement1 [file NIHMS1947908-supplement-Supplement1.docx]

**Supplementory Materials for “Distribution of Select Per- and Polyfluoroalkyl Substances at a Chemical Manufacturing Plant”**

Brian A. Schumacher^a*^, John H. Zimmerman^b^, Alan C. Williams^b^, Christopher C. Lutes^c^, Chase W. Holton^d^, Elsy Escobar^e^, Heidi Hayes^f^, and Rohit Warrier^g^

^a^U.S. EPA Office of Research and Development, Center for Environmental Measurement and Modeling, Ecosystem Processes Division, Athens, GA

^b^U.S. EPA ORD, Center for Environmental Measurement and Modeling, Watershed and Ecosystem Characterization Division, Research Triangle Park, NC.

^c^Jacobs, Cary, NC.

^d^Geosyntec Consultants, Greenwood Village, CO.

^e^Jacobs, Philadelphia, PA.

^f^Eurofins Air Toxics, Folsom, CA.

^g^Research Triangle Institute, International, Research Triangle Park, NC.

*****Corresponding author, Brian Schumacher, phone 1-706-355-8001, schumacher.brian@epa.gov

**The email addresses of all authors:**

**Brian Schumacher:** [schumacher.brian@epa.gov](mailto:schumacher.brian@epa.gov); **John Zimmerman:** [zimmerman.johnh@epa.gov](mailto:zimmerman.johnh@epa.gov); **Alan Williams:** [williams.alan@epa.gov](mailto:williams.alan@epa.gov) ; **Christopher Lutes:** [christopher.lutes@jacobs.com](mailto:christopher.lutes@jacobs.com); **Chase Holton:** [CWHolton@gsi-net.com](mailto:CWHolton@gsi-net.com); **Elsy Escober:** [Elsy.escobar@jacobs.com](mailto:Elsy.escobar@jacobs.com); **Heidi Hayes:** [Heidi.hayes@eurofinset.com](mailto:Heidi.hayes@eurofinset.com); **Rohit Warrier:** rwarrier@rti.org

This SM file contains: 15 pages with 5 Tables and 2 Figures.

# List of Acronyms and Abbreviations

AFFF Aqueous Film-Forming Foams

BGS Below Ground Surface

CCV Continuing Calibration Verification

FTOH Fluorotelomer Alcohol

HDPE High Density Polyethylene

HLC Henry’s Law Constant (in atm m3/mol)

LCS/LCSD Laboratory Control Sample/Laboratory Control Sample Duplicate

MRM Multiple Reaction Monitoring

MTBE Methyl tert-Butyl Ether

PFAS Per- and Polyfluoroalkyl Substances

PFBA Perfluorobutanoic Acid

PFCAs Perfluoroalkyl Carboxylic Acids

PFHP Perfluoro-1-Heptene

PFHpA Perfluoroheptanoic Acid

PFHxA Perfluorohexanoic Acid

PFOA Perfluorooctanoic Acid

PFPeA Perfluoropentanoic Acid

PID Photoionization Detector

RLs Reporting Limits

QC Quality Control

SPE Solid Phase Extraction

TOC Total Organic Carbon

**Table S1. Soil Characteristics and Field Notes at the New Jersey Site.**

| **Location** | **pH** | **TOC** | **Sand** | **Silt** | **Clay** | **Texture** | **Moisture Content** | **Field Notes** |
| --- | --- | --- | --- | --- | --- | --- | --- | --- |
|  |  | % | % | | |  | % |  |
| A | 7.31 | 1.16 | 71.2 | 14.4 | 14.4 | Sandy loam | 10.6 | 0 to 15 cm - coarse sand with cobbles  46 to 106 cm - coarse brown sand  106 cm - water table |
| B | 7.06 | 1.98 | 76.6 | 14.2 | 9.2 | Sandy loam | 10.7 | 122 cm - refusal |
| C | 7.85 | 7.96 | 69.7 | 22.2 | 8.1 | Sandy loam | 15.0 | 0 to 30 cm - coarse sand with cobbles  30 to 46 cm - coarse dark sand  46 to 61 cm - pale light brown silt, cohesive non-plastic trace clay, reddish/brown redox lamination features.  61 – 76 cm - redox features increase  91 cm - refusal |
| D | 6.50 | 0.14 | 83.3 | 6.3 | 10.4 | Loamy Sand | 8.1 | 0 to 76 cm - fine to medium sand  107 cm - white silt layer with redox features  122 cm - water table |

**Table S2. Target PFAS with their Associated Chemical Properties.**

| CASRN | Short Name | Compound Class^†^ | Compound | VP^‡^  20-25°C (mm Hg) | HLC Minimum (unitless) | HLC Average (unitless) | HLC Maximum (unitless) | pKa  Minimum  (unitless) | pKa  Maximum  (unitless) |
| --- | --- | --- | --- | --- | --- | --- | --- | --- | --- |
| 4151-50-2 | N-EtFOSA | FOSA | N-Ethylperfluoro-1-octanesulfonamide | 0.08 | 6.83E-09 | 3.56E+03 | 1.91E+04 | -- | -- |
| 31506-32-8 | N-MeFOSA | FOSA | N-Methylperfluoro-1-octanesulfonamide | 0.12 | 7.77E-09 | 3.85E+03 | 2.34E+04 | -- | -- |
| 2043-47-2 | 4:2 FTOH | FTOH | 2-Perfluorobutyl ethanol | 6.2 | 1.09E-07 | 2.56 | 18.2 | -- | -- |
| -- | 5:2 sFTOH | sFTOH | 1-Perfluoropentyl ethanol | -- | -- | -- | -- | -- | -- |
| 647-42-7 | 6:2 FTOH | FTOH | 2-Perfluorohexyl ethanol | 2.2 | 1.13E-08 | 18.8 | 251 | -- | -- |
| 24015-83-6 | 7:2 sFTOH | sFTOH | 1-Perfluoroheptyl ethanol | -- | -- | -- | -- | -- | -- |
| 678-39-7 | 8:2 FTOH | FTOH | 2-Perfluorooctyl ethanol | 0.23 | 8.87E-09 | 2.57E+02 | 3.47E+03 | 14.19 | 14.19 |
| -- | 9:2 sFTOH | sFTOH | 1-Perfluorononyl ethanol |  | -- | -- | -- | -- | -- |
| 865-86-1 | 10:2 FTOH | FTOH | 2-Perfluorodecyl ethanol | 0.06 | 1.61E-08 | 5.46E+03 | 4.68E+04 | -- | -- |
| -- | 11:2 sFTOH | sFTOH | 1-Perfluoroundecyl ethanol | -- | -- | -- | -- | -- | -- |
| 39239-77-5 | 12:2 FTOH | FTOH | 2-Perfluorododecyl ethanol | -- | -- | -- | -- | -- | -- |
| -- | 13:2 sFTOH | sFTOH | 1-Perfluorotridecyl ethanol | -- | -- | -- | -- | -- | -- |
| 375-22-4 | PFBA | PFCA | Perfluorobutanoic acid | 3.7 | 5.00E-04 | 2.51E-01 | 2 | 0.08 | 0.4 |
| 375-85-9 | PFHpA | PFCA | Perfluoroheptanoic acid | 0.6 | 9.07E-09 | 12.6 | 100 | -0.19 | -0.15 |
| 307-24-4 | PFHxA | PFCA | Perfluorohexanoic acid | 0.91 | 1.03E-08 | 3.38 | 26.9 | -0.16 | 0.7 |
| 335-67-1 | PFOA | PFCA | Perfluorooctanoic acid | 0.35 | 8.26E-09 | 28.9 | 372 | -0.5 | 3.8 |
| 2706-90-3 | PFPeA | PFCA | Perfluoropentanoic acid | 0.92 | 1.32E-08 | 1.81 | 7.24 | -0.1 | 0.4 |
| 375-73-5 | PFBS | PFSA | Perfluorobutanesulfonic acid | 0.21 | 1.24E-08 | 2.62 | 10.5 | 0.14 | 0.3 |
| 355-46-4 | PFHxS | PFSA | Perfluorohexanesulfonic acid | 8.2E-09 | 8.01E-09 | 35.3 | 141 | 0.14 | 0.3 |
| 1763-23-1 | PFOS | PFSA | Perfluorooctanesulfonic acid | 2.5E-06 | 7.56E-10 | 150 | 1950 | 0.14 | 1 |

† - FOSA = perfluorooctane sulfonamide, PFCA = perfluoroalkyl carboxylic acid, PFSA = perfluorosulfonic acid.

‡ - VP = Vapor pressure, HLC = Henry’s Law Constant, °C = degrees Celsius, Pa = Pascals.

* HLC = Henry’s Law Constant is defined as the relative concentrations of a compound between an aqueous solution and gas phase at equilibrium and is reported in its dimensionless form or as a unitless fraction. pKa is the negative logarithm of the equilibrium acid dissociation constant, reported as a unitless fraction. Minimum, average and maximum values of HLC and values of VP are summarized from values reported by ITRC (<https://pfas-1.itrcweb.org/>), last updated October, 2021. Average Henry’s law constants, minimum and maximum pKa values were used in equilibrium partitioning calculation discussion. Among fluorotelomer alcohols, pKa values have been reported only for 8:2 FTOH (Gomis et al., 2015). pKa values for 4:2 FTOH, 6:2 FTOH and 10:2 FTOH were assumed to be similar to 8:2 FTOH in equilibrium partitioning calculations.

Reference:

Gomis, M.I., Z. Wang, M. Scheringer, I.T. Cousins. 2015. A modeling assessment of the physicochemical properties and environmental fate of emerging and novel per- and polyfluoroalkyl substances. Sci. Total Environ*.* 505, 981-991.

**Table S3. Quality Control Acceptance Criteria by Method.**

Modified TO-17 PFAS Soil Gas Compound Reporting Limits and QC Acceptance Criteria

| Analytes | Reporting Limit (ng total per sample tube) | Acceptance Criteria | | | |
| --- | --- | --- | --- | --- | --- |
|  |  | ICAL (%RSD) | | LCS (% R) | CCV (%D) |
| 2-Perfluorobutyl ethanol (4:2 FTOH) | 0.40 | 30 | | 70 – 130 | 30 |
| 2-Perfluorohexyl ethanol (6:2 FTOH) | 0.40 | 30 | | 70 – 130 | 30 |
| 1-Perfluoroheptyl ethanol (7:2sFTOH) | 0.40 | 30 | | 70 - 130 | 30 |
| 2-Perfluorooctyl ethanol (8:2 FTOH) | 0.40 | 30 | | 70 – 130 | 30 |
| 2-Perfluorodecyl ethanol (10:2 FTOH) | 0.40 | 30 | | 70 – 130 | 30 |
| Perfluorobutanoic acid (PFBA) | 0.10 | 30 | | 70 – 130 | 30 |
| Perfluoropentanoic acid (PFPeA) | 0.10 | 30 | | 70 – 130 | 30 |
| Perfluoroheptanoic acid (PFHpA) | 0.10 | 30 | | 70 – 130 | 30 |
| Perfluorohexanoic acid (PFHxA) | 0.10 | 30 | | 70 – 130 | 30 |
| Perfluorooctanoic acid (PFOA) | 0.10 | 30 | | 70 – 130 | 30 |
| n-Methylperfluoro-1-octanesulfonamide (n-MeFOSA) | 0.40 | 30 | | 70 – 130 | 30 |
| n-Ethylperfluoro-1-octanesulfonamide (n-EtFOSA) | 0.40 | 30 | | 70 – 130 | 30 |
| Internal Standards | | | | | |
| Analyte | CCV IS % Recovery | | Sample IS % Recovery | | |
| 2-Perfluorohexyl[1,1-2H2]-[1,2-13C2]ethanol | 60 – 140 | | 60 – 140 | | |
| Perfluoro-n-[1234-13C4]octanoic acid | 60 – 140 | | 60 – 140 | | |

Summary of calibration and QC procedures for Modified Method TO-17 soil gas

| QC Check | Minimum Frequency | Acceptance Criteria | Corrective Action |
| --- | --- | --- | --- |
| Multi-point Calibration (minimum of 5 points) | Prior to sample analysis. | <30%RSD | Correct problem then repeat initial calibration curve. |
| Initial Calibration Verification (ICV) | Second Source standard analyzed after each initial calibration curve. | 70-130% | Check the system and reanalyze the standard. Re-prepare the standard if necessary. Re-calibrate the instrument if primary standard preparation is found to be in error. |
| Continuing Calibration Verification (CCV) | At the start of each 24-hour clock after the tune check. | <30%D | Maintenance is performed and the CCV test repeated. If the system still fails the CCV, perform a new 5-point calibration curve. |
| Laboratory Control Sample (LCS) | Second source standard analyzed in each daily batch | 70-130% | Check the system and reanalyze the standard. Re-prepare the standard if necessary. Re-calibrate the instrument if primary standard preparation is found to be in error. |
| Laboratory Control Spike Duplicate (LCSD) | Once per analytical batch | <25%RPD | Verify accuracy of standard. Re-prepare LCSD if necessary.  If calibration curve and/or system is found to be out of control, perform maintenance and re-calibrate. |
| Laboratory Blank | After the CCV and before the samples. | Results less than the laboratory RL. | Inspect the system and reanalyze the blank. |
| Internal Standard (IS) | As each QC sample and sample are being loaded. | CCVs: area counts 60-140%, Retention time (RT) within 20 sec of mid-point in ICAL.  Blanks and samples:  RT must be within ±0.33 minutes of the RT in the CCV. The IS area must be within ±40% of the CCV’s IS area for the blanks and samples. | CCV: Inspect and correct system prior to sample analysis.  Blanks: Inspect the system and reanalyze the blank.  Samples: Investigate the problem by verifying the instrument is in control by running a lab blank. Narrate and flag associated data. If recovery exceedance is due to matrix, analyze back up sample(s) if available, utilizing higher split and/or analyzing lower volume sample. |
| Field Blank | Collected at a frequency of 5% of samples. | Artifact levels should be less than the reporting limit or less 10% of the mass measured on the sampled tubes, whichever is greater. | Flag associated results and evaluate tube conditioning and storage procedures. |
| Field Duplicates** | Collected at a frequency of 5% of samples. | %RPD < 50% | Narrate discrepancy. |

FTOH Analytes Control Limits, Detection Limit, and Reporting Limits

| Analyte | CASRN | LCS Lower Limit^†^ | LCS Upper Limit | RPD | MDL | RL | Units |
| --- | --- | --- | --- | --- | --- | --- | --- |
| 10:2 FTOH-2-Perfluorodecyl ethanol | 865-86-1 | 62 | 113 | 30 | 0.3 | 1 | ug/L |
| 4:2 FTOH-2-Perfluorobutyl ethanol | 2043-47-2 | 41 | 92 | 30 | 0.1 | 1 | ug/L |
| 6:2 FTOH-2-Perfluorohexyl ethanol | 647-42-7 | 48 | 109 | 30 | 0.2 | 1 | ug/L |
| 7:2 FTOH-1-Perfluoroheptyl ethanol | 24015-83-6 | 70 | 130 | 30 | 0.2 | 1 | ug/L |
| 8:2 FTOH-2-Perfluorooctyl ethanol | 678-39-7 | 51 | 112 | 30 | 0.2 | 1 | ug/L |

† LCS = laboratory control sample. RPD = relative percent difference. MDL = method detection limit. RL = reporting limit.

Calibration and QC Procedures for Method 537

| Instrument | Calibration Procedure | Frequency of Calibration | Acceptance Criteria | Corrective Action (CA) |
| --- | --- | --- | --- | --- |
| LC/MS/MS PFAS Compounds in Water | Mass Calibration | Initially, annually, and after performing major maintenance | Per manufacturer specifications | NA |
|  | Tuning of LC/MS/MS | When masses fall outside ± 0.5 amu of true masses | Within 0.5 amu of true value | Retune and verify. If tuning fails acceptance criteria, perform a mass calibration and repeat the tune check. |
|  | Mass Spectral Acquisition Rate | Each analyte, labeled analyte, and injection internal standard | A minimum of 10 spectra scans are acquired across each chromatographic peak | Not Applicable |
|  | Initial calibration with a minimum 5 points | After continuing calibration fails | S/N ratio ≥10:1 for all ions used for quantification. Linear or non-linear calibrations must have a r^2^ ≥0.99. Must use at least 6 points for a quadratic. Analytes must be within 70-130% of their true value for each calibration standard with the exception of the lowest calibration point which must be within 50-150% of the true value. | Perform more aggressive instrument maintenance and recalibrate |
|  | MDL^†^ standard | After each initial calibration | All compounds must be detected | Repeat ICAL procedure prior to analyzing samples. Repeat maintenance if needed. |
|  | ICV Standard | Once with every ICAL | Within ±30% of their true value | Reanalyze the ICV and samples associated with the non-compliant ICV. If ICV fails again do system maintenance, recalibrate, and reanalyze samples. |
|  | LOQ Verification | Quarterly | Within 50% of true value | Reprep and reanalyze LOQ. |
|  | CCV Standard | Every 10 samples and at the end of a sequence | Alternate analyte concentrations range from the LOQ to the mid-level of the calibration curve. Recover within ±30% of their true value | Reanalyze CCV in duplicate immediately. If both pass, samples can be report. If either fails or if immediate reanalysis of CCV in duplicate cannot be performed all samples since acceptable CCV must be reanalyzed. If the CCV fails high any associated samples that are ND can be reported. |

† MDL = method detection limit. ICAL = initial calibration. ICV = initial calibration verification. LOQ = limit

of quantitation. CCV = continuing calibration verification. ND = not detected.

QC Samples for Method 537

| QC Sample | Frequency/Number | Method/SOP QC Acceptance Limits | Corrective Action | Data Quality Indicator (DQI) | Measurement Performance Criteria |
| --- | --- | --- | --- | --- | --- |
| Method blanks | 1 per prep batch of up to 20 samples | No analytes detected >1/10 the amount measured in any sample. | Reanalyze to confirm detections. If detects confirm reextract samples that are not ND or not >10x the blank value | Accuracy/Laboratory Contamination | No analytes detected >1/10 the amount measured in any sample |
| MS/MSD^†^ | 1 per prep batch of up to 20 samples | Laboratory statistical limits | Flag outliers | Accuracy/Bias/Precision | Results within acceptance limits |
| LCS/LCSD | 1 per prep batch of up to 20 samples | Laboratory statistical limits | Reanalyze LCS and associated samples. Analytes in the LCS that fail high and are ND in the samples can be reported. All others are re-extracted. | Accuracy/Bias/Precision | Results within acceptance limits |
| Isotopically Labeled Extraction Standards | Per sample (including MS/MSD, LCS, and blanks) prior to preparation | Within laboratory statistical limits | If fails for QC sample, but the native compounds are within specification, report data. If fails for sample, reextract and reanalyze and/or consult a supervisor for course of action. | Accuracy | Results within acceptance limits |
| Isotopically Labeled Injection Standards | Per sample (including MS/MSD, LCS, and blanks) prior to preparation | Absolute area must be -50% to +50% of the average areas measured during the ICAL | Analyze a second aliquot of the extract. If none remains, reanalyze first aliquot and/or consult a supervisor to determine course of action | Accuracy | Results within acceptance limits |

†MS/MSD = matrix spike/matrix spike duplicate. LCS/LCSD = laboratory control sample/laboratory control sample duplicate. ICAL = initial calibration.

**Table S4. Spreadsheets and References for PFAS Equilibrium Calculations by Location.**

Location A

Location B

Location C

Location D

Table S5. Laboratory 2 FTOH Concentrations in Soil for New Jersey Field Event 1 (July 2021).

| **Chemical Identification** | | | **Laboratory 2 Soil Concentration - Sample Location A (ng/g)** | **Laboratory 2 Soil Concentration - Sample Location B (ng/g)** | **Laboratory 2 Soil Concentration - Sample Location B (ng/g) Dup** | **Laboratory 2 Soil Concentration - Sample Location C (ng/g)** | **Laboratory 2 Soil Concentration - Sample Location D (ng/g)** |
| --- | --- | --- | --- | --- | --- | --- | --- |
| **Chemical Class** | **Chemical Name** | **Substance Acronym** |  |  |  |  |  |
| **Perfluorinated telomer alcohols (FTOHs)** | 2-perfluorobutyl ethanol | 4:2 FTOH | 0.05^*^ | 0.06 | 0.38 | 0.12 | 0.09 |
|  | 1-perfluoropentyl ethanol | 5:2 sFTOH | 0.15 | <0.22 | <0.22 | 4.78 | <0.22 |
|  | 1-perfluoroheptyl ethanol | 7:2 sFTOH | <0.13 | <0.13 | <0.13 | 4.91 | <0.13 |
|  | 2-perfluorohexyl ethanol | 6:2 FTOH | 1.45 J^†^ | 3.24 J | 1.71 | 2.38 | <0.21 |
|  | 2-perfluorooctyl ethanol | 8:2 FTOH | 1.01 J | 2.92 J | 6.71 J | 3.56 | 0.48 J |
|  | 2-perfluorodecyl ethanol | 10:2 FTOH | 4.48 J | 20.85 | 3.44 J | 36.15 | <0.26 |

* value is average of replicate analyses.

^†^ J – estimated value.

**Figure S1. Sampling Locations for the New Jersey Site.**

General Groundwater Flow Direction


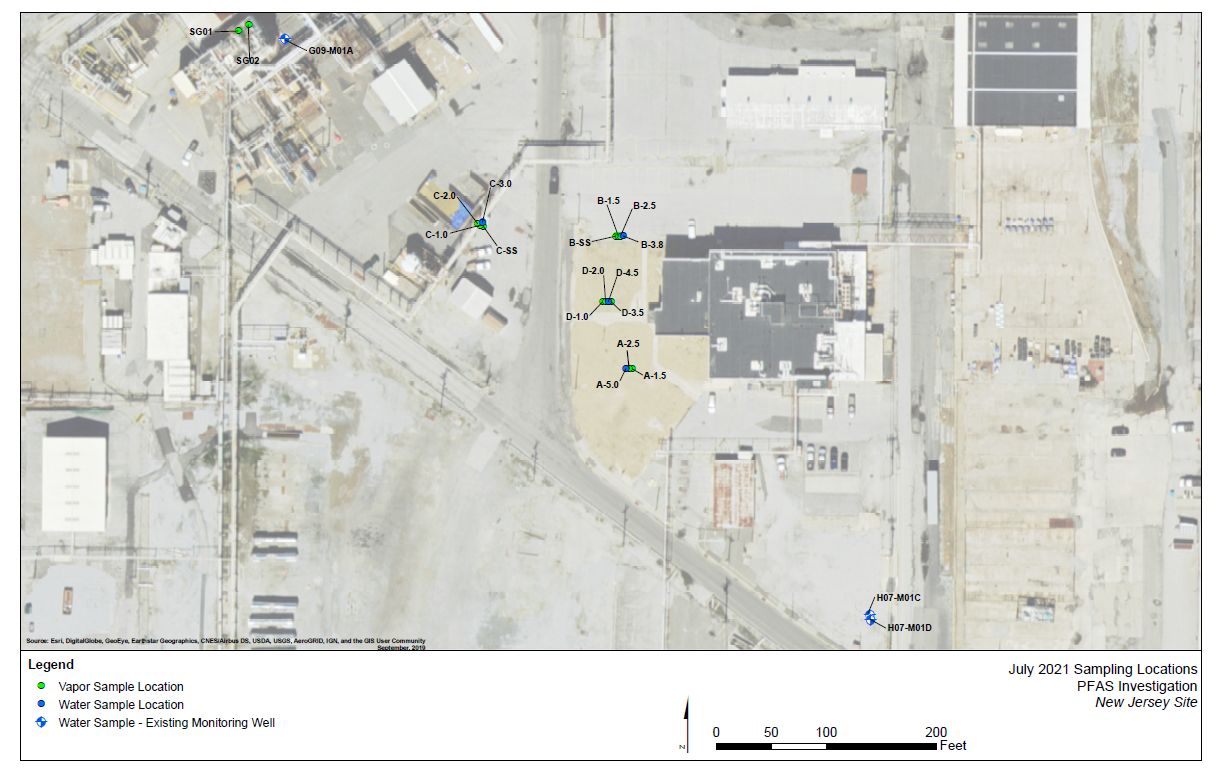


**Figure S2. Subslab and Soil Gas Sampling System.**


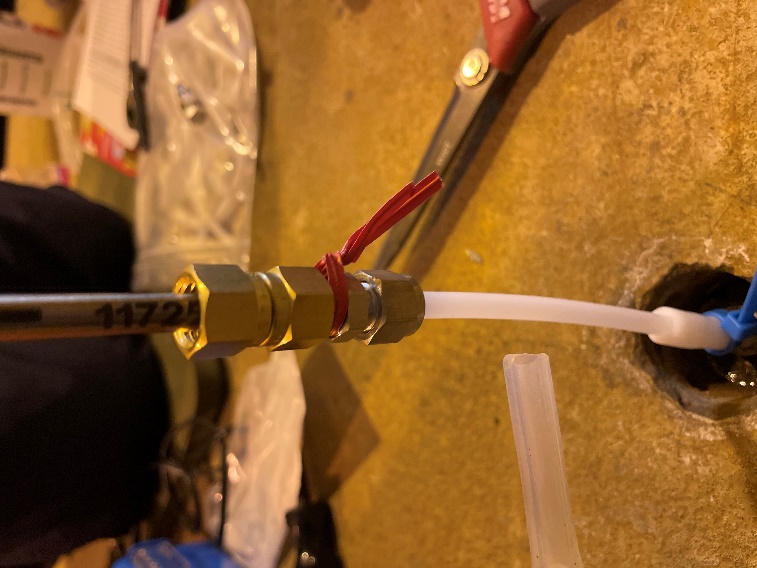


Location of filter if in use.

Sampling line to sublab or soil gas probe

Connection to subslab port

ATD tube

Syringe used to pull soil or subslab gas through ATD tube


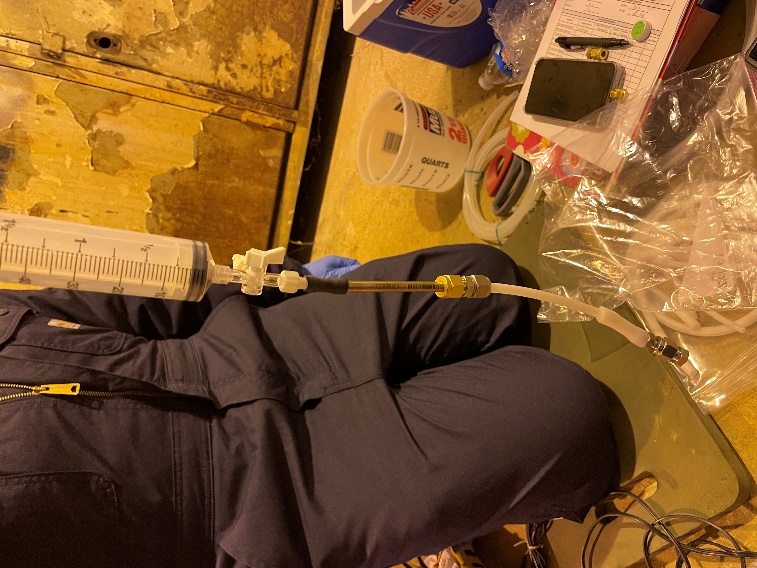


ATD Tube

Sampling line to sublab or soil gas probe

System prior to connection of subslab or soil gas port.
